# Expanded Geographical Distribution of *Coleomegilla maculata lengi* (Coleoptera: Coccinellidae) in North America

**DOI:** 10.3390/insects15050305

**Published:** 2024-04-25

**Authors:** Louis Hesler, Mathew Brust

**Affiliations:** 1 North Central Agricultural Research Laboratory, Agricultural Research Service, USDA, Brookings, SD 57006, USA; 2 Department of Biology, Chadron State College, Chadron, NE 69337, USA; mbrust@csc.edu

**Keywords:** Coccinellini, persistence, regional collections

## Abstract

**Simple Summary:**

*Coleomegilla maculata lengi* is an important insect predator in North American agroecosystems. Collection records reveal that the reputed range for *C. maculata lengi* has been substantially underestimated. New records indicate an expanded geographic range, including the recent expansion of *C. maculata lengi* in central North America and a new state record for Wyoming, USA. The expanding geographic range of *C. maculata lengi* contrasts with that of several species of lady beetles native to North America.

**Abstract:**

Several species of lady beetle native to North America have declined in abundance in the last few decades, often accompanied by contractions in their geographic ranges. *Coleomegilla maculata lengi* is a lady beetle native to North America that is an important predator of pests in various agroecosystems. Its reported range spans the eastern half of the USA, with no sustained decline in abundance or contraction of its range reported. Indeed, we recently collected several individuals of this lady beetle in central USA roughly 500 km beyond the western edge of its reputed range. We hypothesized that new records could indicate either that previous range characterization failed to include pre-existing collection records further west or that *C. maculata lengi* has recently expanded its geographic range. To test these hypotheses, we searched several institutional insect collections and digital databases for records and found many earlier records of *C. maculata lengi* beyond its reputed geographic range, clearly showing that the previous characterization of its geographic distribution in North America was substantially underestimated. In addition, we report a new state record of *C. maculata lengi* from Wyoming, USA, that further indicates its geographic range expansion in North America. We discuss new records of *C. maculata lengi* in light of declines in native coccinelline lady beetle species in North America.

## 1. Introduction

Lady beetles are important predators of arthropod pests, such as aphids, mealybugs, scale insects, and mites [1]. Over the last few decades, several of the larger native species of aphidophagous lady beetles (Coleoptera, Coccinellidae, Coccinellinae, Coccinellini) have decreased in abundance, sometimes becoming locally rare or even locally extinct at various sites in America north of Mexico [2,3,4,5,6,7,8,9]. Temporally, these declines started roughly around the time that a small number of non-native coccinelline species were established and spread within the region [2,3,4,5,6,10,11,12].

**Figure 1:**
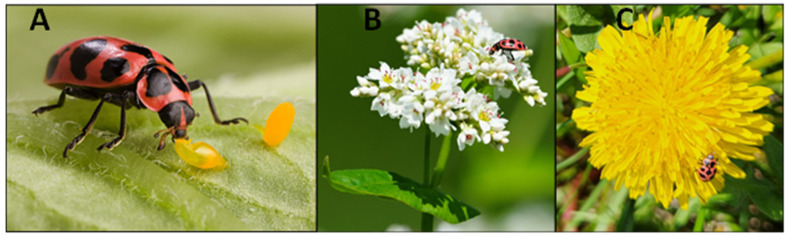
*Coleomegilla maculata lengi* consuming eggs of Colorado potato beetle, *Leptinotarsa decemlineata* (**A**); pollen of buckwheat, *Fagopyrum esculentum* (**B**); and dandelion, *Taraxacum officianale* (**C**). Photo credits: Peggy Greb (**A**), Jim Eklund (**B**), and Eric Beckendorf (**C**), all USDA-ARS.

As native lady beetles have declined in North America, researchers have called for ongoing studies to monitor and assess their populations [2,13,14]. During recent surveys of coccinellines in central USA, we sampled the native lady beetle *Coleomegilla maculata lengi* Timberlake (Figure 1) from western South Dakota (Pennington County) [15] and western Nebraska (Dawes and Sioux counties) [16].

**Figure 2:**
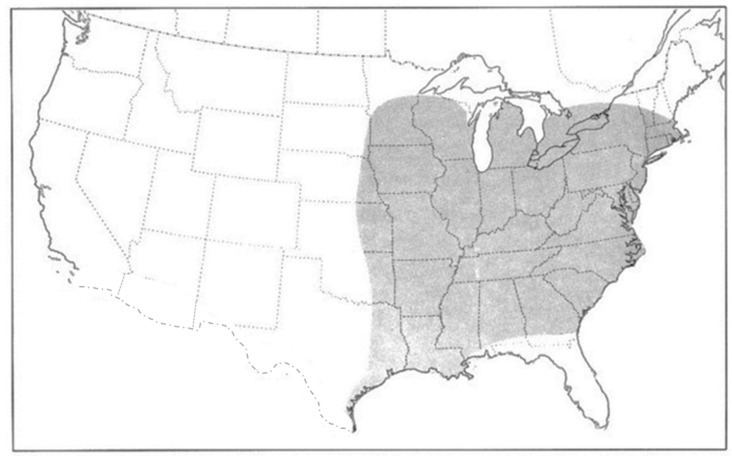
Distribution of *Coleomegilla maculata lengi* (gray shading, eastern North America; Gordon 1985). Original figure used by permission of the New York Entomological Society.

*Coleomegilla maculata lengi* is one of three subspecies of *Coleomegilla maculata* (DeGeer) native to America north of Mexico [17]. *Coleomegilla maculata strenua* (Casey) is distributed along the southern border of the United States from California to Texas and *C. m. fuscilabris* (Mulsant) is distributed in Florida, USA, and along the Gulf Coast to Louisiana, USA [17]. The geographic distribution of *C. maculata lengi* is projected to extend from the Great Lakes region of Canada and the eastern half of the United States south to the coastal areas of Louisiana and Texas along the Gulf of Mexico [17] (Figure 2). The recent collections of *C. maculata lengi* from western Nebraska and western South Dakota were unexpected as they were roughly 500 km beyond its purported westernmost distribution [17].

Three hypotheses could explain the presence of *C. maculata lengi* in far western Nebraska and western South Dakota. First, these records of *C. maculata lengi* could demonstrate a recent western geographic range expansion and provide a counterexample to the trend of contracted geographical ranges for some other North American coccinelline species. Alternatively, western collection records could indicate the establishment of a disjunct population of *C. maculata lengi*. On the other hand, western records may indicate an underestimate of the distribution range of *C. maculata lengi* by Gordon [17]. For instance, McAlpine et al. [18] pointed out additional records of *C. maculata lengi* from the state of Maine, USA, from as early as 1907, indicating that its northeastern distribution had been underestimated [17]. In addition, recent records of *C. maculata lengi* from southeastern Canada also suggest an underestimate of its northeastern range, a range expansion, or both [18,19]. In light of the new records for *C. maculata lengi* across North America, we searched insect collections and collection databases for records of *C. maculata lengi* from the United States and Canada to assess evidence for the three competing hypotheses about this lady beetle’s presence in western South Dakota and western Nebraska.

## 2. Materials and Methods

To test the various hypotheses, we searched several institutional insect collections and digital databases for records of *C. maculata lengi*. Collections housed at the following institutions were searched:

- CSCC: Chadron State College Insect Collection
- ESUW: University of Wyoming Insect Collection
- KSUC: Kansas State University
- NDSU: North Dakota Insect Research Collection
- SDSU: Severin–McDaniel Insect Research Collection
- SEMC: University of Kansas, Snow Entomological Museum
- UNSM: University of Nebraska State Museum
- NCARL: USDA-ARS North Central Agricultural Research Laboratory Collection

In addition, we searched the online records of *C. maculata lengi* in the Symbiota Collections of Arthropods Network (SCAN) [20] and the Global Biodiversity Information Facility [21], which yielded specimen records from the following institutions:

- FMNH: Field Museum of Natural History Collection of Insects, Arachnids, and Myriapods
- NMSU: New Mexico State Collection of Arthropods

Collections were searched for records of *C. maculata lengi* within an area bounded roughly by 37–50° N latitude and 97–107° W longitude that lay adjacent to its reputed westernmost distribution [17]. As such, the search area included roughly the western three-quarters of the states of Kansas, Nebraska, South Dakota, and North Dakota; the eastern tier of counties in Colorado, Wyoming, and Montana; and southern portions of the provinces of Manitoba and Saskatchewan, Canada. We conservatively delimited our searches geographically to roughly the southern border of Kansas because of the potential for confounding subspecies of *C. maculata* and nomenclatural ambiguity in records from more southern locations [17, personal observations]. Although Gordon [17] did not specify a cut-off date for records used in his treatise of American coccinellids north of Mexico, we limited our searches temporally to records of 1979 and earlier.

Specimen records were categorized by year and county. A subset of the collection records was developed so that only the earliest records of *C. maculata lengi* for individual counties were retained. The earliest records per county were grouped by decade (e.g., 1910–1919) and used to generate a county-level map depicting the first occurrences of *C. maculata lengi* by decade (MapChart v.3.0, https://www.mapchart.net/usa-counties.html, accessed 27 February 2024). The map provided a visual aid to evaluate the three hypotheses regarding the recent finds of *C. maculata lengi* in western Nebraska and western South Dakota, namely, (1) specimens collected west of the reputed range limit many years before 1980 would demonstrate a lack of their accounting in the development of the distribution map [17]; (2) collection records confined to later dates, i.e., mainly the 1970s, and within roughly 150 km of the reputed western range limit would suggest a range expansion that might not have been realized in a 1985 estimate of the beetle’s distribution [17]; and (3) a lack of collection records from points between the eastern and western edges of Nebraska and South Dakota, regardless of date, would suggest a disjunct population far beyond the western edge of the beetle’s main distribution. Ad hoc comparisons of the distributions of collection records were made using a two-sample proportions test [22].

## 3. Results

### 3.1. Collection Records, 1910–1979

**Figure 3:**
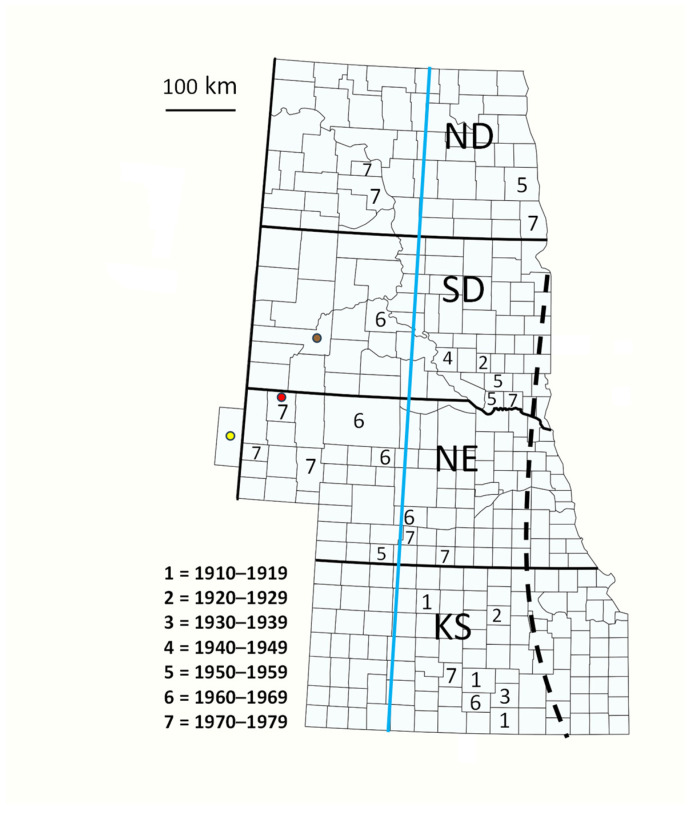
Counties in central USA with collection records of *Coleomegilla maculata lengi*. Numbers depict decades of the first pre-1980 collection record in a county west of the reputed distribution. Dots depict counties with recent collection records: brown, 2008, Pennington Co., SD; red, 2010 and 2011, Dawes Co., NE; and yellow, Goshen Co., WY, 2014. Dashed line indicates western edge of reputed range in 1985. Blue line demarcates eastern and western portions of records area. ND = North Dakota, SD = South Dakota, NE = Nebraska, KS = Kansas, WY = Wyoming.

Altogether, we found 101 specimen records of *C. maculata lengi* from Kansas, Nebraska, South Dakota, and North Dakota at locales west of the reputed 1985 distribution range with collection dates that extended from 1910 to 1978. There were 35 distinct location–dates during that period, each comprising one to 52 specimens (Appendix A). The 35 location–dates comprised 26 counties in the four states (Figure 3). No records of *C. maculata lengi* from 1910 through 1979 were found in states further west, i.e., Colorado, Wyoming, and Montana. Though patchy, records were obtained from several counties in North Dakota, South Dakota, the western three-quarters of Nebraska, and the western two-thirds of Kansas. The proportions of new county records since the midpoint year (1945) differed significantly between counties in the eastern (60 percent) and western (100 percent) portions of the search area (blue line, Figure 3; Z = 2.39, *p* = 0.008), but did not differ as a latitudinal dichotomy between records from North Dakota and South Dakota (90 percent) versus Nebraska and Kansas (69 percent) (Z = 1.25, *p* = 0.10).

### 3.2. New Distribution Record

In addition, while perusing collection data for *C. maculata lengi*, we came across a recent record from the state of Wyoming, which constitutes a **new state record** for this lady beetle: USA, WYOMING: Goshen County, Lingle, Ag Exp Station, BII-1, 22 July 2014, M. Benander. The collection site was located in southeastern Wyoming in a county that borders the western edge of Nebraska.

## 4. Discussion

The collection data in Appendix A support the hypothesis that records from North Dakota, South Dakota, and central and western Nebraska and Kansas were not accounted for in the development of Gordon’s [17] distribution map of *C. maculata lengi.* Of particular note, a 1978 record of *C. maculata lengi* from Dawes County, Nebraska (Appendix A), pre-dates recent collection records [16] from that county by >30 years. Though patchy, the records from 1910 to 1978 were distributed at various points across the four-state area, and counter the alternative hypothesis of a disjunct distribution of *C. maculata lengi* in western Nebraska and southwestern South Dakota. Furthermore, our collection data do not support the hypothesis of an abrupt westward range expansion of *C. maculata lengi* because records spanned the period of 1910 to 1978, although evidence for a relatively recent westward range expansion was supported by records west of 99.5° longitude occurring exclusively since 1956. Due to the lack of previous records from far western South Dakota and adjacent states to its west, the 2008 record of *C. maculata lengi* from Pennington County [15] and the 2014 record from Wyoming in this study indicate a continued westward range expansion of *C. maculata lengi*. The straight-line distance between the site of the earliest westernmost record of *C. maculata lengi* from Medora, Kansas, in 1910 and the Lingle, Wyoming, record in 2014 is 711 km. Based on the time and distance between those two records, one can surmise that *C. maculata lengi* has expanded its westward range at a rate of 6.8 km/yr. We did not find rate estimates of range expansion for lady beetle species native to North America, but invasive lady beetles have spread throughout North America at various rates, ranging from 15 km/year for *Coccinella undecimpunctata* to 415 km/yr for *C. septempunctata* [23].

Our findings of western records of *C. maculata lengi* are consistent with additional studies that have shown an underestimation of its previously purported distribution and evidence of geographic expansion in other areas of its range. For instance, McAlpine et al. [18] noted that the omission of *C. maculata lengi* specimens from southern and central Maine, USA, led to an underestimation of its northeastern range. Additional collection records from northern Maine and New Brunswick, Canada, may reflect either a lack of coccinellid collecting activities in that region or northward expansion [18]. Additional records of *C. maculata lengi* in the Insectarium de Montréal (IMQC) document the presence of *C. maculata lengi* in Quebec, Canada, as early as 1975 [20] and provide another instance of range underestimation. Additional northward range expansion by *C. maculata lengi* in central Canada was documented in 1988 collection records from southern Manitoba [24].

Reasons for discrepancies between the purported distribution of *C. maculata lengi* [17] and the findings of records outside the reputed range may lie in differences in the sources consulted. Gordon [17] borrowed specimens and consulted with individuals from at least 21 institutions and, remarkably, produced distribution maps or comments on the geographic ranges that accompanied descriptions of all 475 species in his iconic work on the Coccinellidae of America north of Mexico. However, there is no evidence that Gordon [17] investigated specimens from any of the collections that we consulted for central North American records of *C. maculata lengi*, collections from the Maine State Museum regarding specimens from southern and central Maine, or the IMQC specimens from Quebec.

Analogous examples of incomplete distributional information have been discovered for other beetle taxa. For instance, examinations of regional insect collections have exposed underestimates of distributions of long-horned beetles (Cerambycidae) in eastern Canada [25,26]. These underestimates stemmed from researchers who overlooked regional collections in compiling large-scale geographic distributions and from a lack of researchers working with regional collections to publish records that could have been used in distributional compilations [25]. Furthermore, it may be particularly important to search regional collections and databases for specimen records from near or even beyond the reputed limits of a species’ geographic range to test notions about its distribution and increase the likelihood of accurately mapping the distribution.

The need for accuracy has been heightened by an increasing awareness of changes in the abundance and distribution of North American coccinellid in recent decades [6]. For adventive species, many have continued to expand their geographic ranges in North America and, in some cases, have become invasive [17,23,27,28]. In contrast, the abundance of several native coccinellids has decreased and, in some instances, this has been accompanied by apparent contractions in their geographic ranges [2,3,5,7,12,29]. However, *C. maculata lengi* is a clear counterexample of a native lady beetle that has increased in relative abundance in some regions (e.g., New York, USA) [10] and that is undergoing an expansion of its geographic distribution, based on evidence from this study and others [18,24]. The reason(s) for its relative abundance and range expansion is unclear, but Losey et al. [10] noted that *C. maculata lengi* is facultatively pollenivorous (Figure 1) rather than an obligate predator like other coccinellids and that it may, therefore, have been less impacted by competition for food resources with adventive coccinelline species. Follow-up surveys will be needed to delineate the expanding geographic range of *C. maculata lengi*, and additional studies are needed to determine the reasons for its persistence in light of declines in other native coccinellids.

## Data Availability

The data presented in this study are available in the article.

## Appendix A. Pre-1980 Records of *Coleomegilla maculata* lengi from a Four-State Area in Central USA Bounded Roughly by 37–50° N Latitude and 97–107° W Longitude

**State**	**County**	**Locale**	**Date**	**Collector(s)**	**No. of Specimens**	**Collection Codon**
Kansas	Reno	Medora	4 July 1910		1	KSUC
	Rooks		9 August 1912	Francis William	1	SEMC
	Sumner		1916	Raymond Beamer	3	SEMC
	Ottawa	Franklin	1 June 1927	H. Hungerford	1	SEMC
	Sedgwick	Wichita	15 June 1931		1	NMSU
	Sedgwick	Wichita	18 August 1931		1	NMSU
	Ottawa	Franklin	1 June 1956	D. Lang	2	SEMC
	Sedgwick		29 October 1957	J.R. Zimmerman	1	NMSU
	Kingman	Kingman, 8 mi W	10 May 1962	Charles Michener	1	SEMC
	Stafford		August 1978		1	KSUC
Nebraska	Red Willow	7 mi. E. of McCook	27 August 1958	Atyeo and Anderson	1	UNSM
	Dawson		29 May 1968	A. Beltran	52	UNSM
	Dawson		29 May 1968	K. Pruess	1	UNSM
	Cherry	Valentine	23 June 1968	A. Beltran	2	UNSM
	Thomas	Halsey	July 1969		1	UNSM
	Red Willow	McCook	29 May 1971	B.C. Ratcliffe	1	UNSM
	Scotts Bluff	Scottsbluff/	17 April 1974	F. Snocker	1	CSCC
		North Platte River				
	Garden		27 July 1974	W. T. Morgan	1	CSCC
	Gosper	Elwood	18 June 1975	A. F. Newton, M. K. Thayer	1	FMNH
	Franklin		18–24 June 1978		3	UNSM
	Dawes		10 October 1978		1	CSCC
North Dakota	Cass	Fargo, NDAC	7 August 1956	Richard L. Post	1	NDSU
	Cass	Fargo	1957		1	NDSU
	Richland	Walcot Dunes	10 July 1973	Tim L. McCabe	1	NDSU
	Cass	Fargo	27 September 1973	P.K. Lago	1	NDSU
	Richland	Mirror Pool	27 May 1974	P.K. Lago	1	NDSU
	Morton	6 mi E Glen Ullin	1 August 1975	P.K. and B.A. Lago	1	NDSU
	Oliver	2 mi E Hensler	20 July 1978	E.U. Balsbaugh	1	NDSU
South Dakota	Davison	Mitchell	21 August 1929		2	SDSU
	Brule	Chamberlain	14 September 1946		4	SDSU
	Brule	Chamberlain	17 June 1947		1	SDSU
	Bon Homme	Springfield	28 May 1954		4	SDSU
	Hutchinson	Dimock	1 September 1954		1	SDSU
	Stanley	Hayes	2 August 1967		2	SDSU
	Yankton	Yankton	13 May 1974		1	NDSU
	Yankton	Yankton	11 June 1974		1	NDSU

## References

[1] Michaud J.P., Hodek I., van Emden H.F., Honěk A. (2012). Coccinellids in biological control. Ecology and Behaviour of the Ladybird Beetles (Coccinellidae).

[2] Wheeler A.G., Hoebeke E.R. (1995). *Coccinella novemnotata* in northeastern North America: Historical occurrence and current status (Coleoptera: Coccinellidae). Proc Entomol. Soc. Washington.

[3] Elliott N., Kieckhefer R., Kauffman W. (1996). Effects of an invading coccinellid on native coccinellids in an agricultural landscape. Oecologia.

[4] Turnock W.J., Wise I.L., Matheson F.O. (2003). Abundance of some native coccinellines (Coleoptera: Coccinellidae) before and after the appearance of *Coccinella septempunctata*. Can. Entomol..

[5] Alyokhin A., Sewell G. (2004). Changes in a lady beetle community following the establishment of three alien species. Biol. Invasions.

[6] Harmon J.P., Stephens E., Losey J. (2007). The decline of native coccinellids (Coleoptera: Coccinellidae) in the United States and Canada. J. Insect Conserv..

[7] Hesler L.S., Kieckhefer R.W. (2008). Status of exotic and previously common native coccinellids (Coleoptera) in South Dakota landscapes. J. Kansas Entomol. Soc..

[8] Hesler L.S., Beckendorf E.A. (2021). Declining abundance of Coccinellidae (Coleoptera) among crop and prairie habitats of eastern South Dakota, USA. Front. Conserv. Sci..

[9] Bahlai C.A., Colunga-Garcia M., Gage S.H., Landis D.A. (2015). The role of exotic ladybeetles in the decline of native ladybeetle populations: Evidence from long-term monitoring. Biol. Invasions.

[10] Losey J.E., Allee L.L., Stephens E., Smyth R.R., Priolo P., Tyrrell L., Chaskey S., Stellwag L. (2014). Lady beetles in New York: Insidious invasions, erstwhile extirpations, and recent rediscoveries. Northeast. Nat..

[11] Evans E.W. (2017). Fates of rare species under siege from invasion: Persistence of *Coccinella novemnotata* Herbst in western North America alongside an invasive congener. Front. Ecol. Evol..

[12] Petersen M.J., Losey J.E. (2024). Niche overlap with an exotic competitor mediates the abundant niche-centre relationship for a native lady beetle. Divers. Distrib..

[13] Ellis D.R., Prokrym D.R., Adams R.G. (1999). Exotic lady beetle survey in northeastern United States: *Hippodamia variegata* and *Propylea quatuordecimpunctata* (Coleoptera: Coccinellidae). Entomol. News.

[14] Obrycki J.J., Elliott N.C., Giles K.L., Follett P.A., Duan J.J. (2000). Coccinellid introductions: Potential for and evaluation of nontarget effects. Nontarget Effects of Biological Control.

[15] Hesler L.S., Catangui M.A., Losey J.E., Helbig J.B., Mesman A. (2009). Recent records of *Adalia bipunctata* (L.), *Coccinella transversoguttata richardsoni* Brown, and *Coccinella novemnotata* Herbst (Coleoptera: Coccinellidae) from South Dakota and Nebraska. Coleopts. Bull..

[16] Bartlett P.B., Hesler L.S., French B.W., Catangui M.A., Gritzner J.H. (2015). Lady beetle assemblages (Coleoptera: Coccinellidae) in western South Dakota and western Nebraska and detection of reproducing populations of *Coccinella novemnotata*. Ann. Entomol. Soc. Am..

[17] Gordon R.D. (1985). The Coccinellidae (Coleoptera) of America north of Mexico. J. N. Y. Entomol. Soc..

[18] McAlpine D.F., Migneault R., Webster R.P. (2018). *Coleomegilla maculata lengi* Timberlake, 1943 (Coleoptera: Coccinellidae), a native North American lady beetle new to Maritime Canada. J. Acadian Entomol. Soc..

[19] Finlayson C.J., Landry K.M., Alyokhin A.V. (2008). Abundance of native and non-native lady beetles (Coleoptera: Coccinellidae) in different habitats in Maine. Ann. Entomol. Soc. Am..

[20] (2024). SCAN (Symbiota Collections of Arthropods Network). http://symbiota4.acis.ufl.edu/scan/portal/.

[21] (2024). GBIF (Global Biodiversity Information Facility). https://gbif.org.

[22] Zar J.H. (2010). Biostatistical Analysis.

[23] Hemptinne J.-L., Magro A., Evans E.W., Dixon A.F. (2012). Body size and the rate of spread of invasive ladybird beetles in North America. Biol. Invasions.

[24] Wise I.L., Turnock W.J., Roughly R.E. (2001). New records of coccinellid species for the Province of Manitoba. Proc. Entomol. Soc. Manitoba.

[25] McCorquodale D.B., Bondrup-Nielsen S. (2004). Do we know beetles? Lessons from new records of Cerambycidae (Coleoptera) for Nova Scotia. Proc. Nova Scotia Instit. Sci..

[26] Majka C.G., McCorquodale D.B., Smith M.E. (2007). The Cerambycidae (Coleoptera) of Prince Edward Island: New records and further lessons in biodiversity. Can. Entomol..

[27] Schaefer P.W., Dysart R.J., Specht H.B. (1987). North American distribution of *Coccinella septempunctata* (Coleoptera: Coccinellidae) and its mass appearance in coastal Delaware. Environ. Entomol..

[28] Koch R.L., Venette R.C., Hutchison W.D. (2006). Invasions by *Harmonia axyridis* (Pallas) (Coleoptera: Coccinellidae) in the western hemisphere: Implications for South America. Neotropic. Entomol..

[29] Diepenbrock L.M., Fothergill K., Tindall K.V., Losey J.E., Smyth R.R., Finke D.L. (2016). The influence of exotic lady beetle (Coleoptera: Coccinellidae) establishment on the species composition of the native lady beetle community in Missouri. Environ. Entomol..

